# A gnotobiotic growth assay for *Arabidopsis* root microbiota reconstitution under iron limitation

**DOI:** 10.1016/j.xpro.2020.100226

**Published:** 2020-12-15

**Authors:** Christopher J. Harbort, Masayoshi Hashimoto, Haruhiko Inoue, Paul Schulze-Lefert

**Affiliations:** 1 Department of Plant Microbe Interactions; Max Plank Institute for Plant Breeding Research, 50829 Cologne, Germany; 2 Graduate School of Agricultural and Life Sciences, The University of Tokyo, 113-8657 Tokyo, Japan; 3 Institute of Agrobiological Sciences, National Agriculture and Food Research Organization (NARO), 305-8602 Tsukuba, Japan; 4 Japan Science and Technology Agency - PRESTO, Honcho, Kawaguchi, Saitama 332-0012, Japan; 5 Cluster of Excellence on Plant Sciences (CEPLAS), Max Planck Institute for Plant Breeding Research, 50829 Cologne, Germany

**Keywords:** Metabolism, Model Organisms

## Abstract

We present a gnotobiotic system for microbiota reconstitution on *Arabidopsis thaliana* under contrasting iron availability. This system induces iron starvation in plants by providing an unavailable form, mimicking conditions in alkaline soils. Inoculation of taxonomically diverse bacteria reconstitutes plants with a synthetic microbiota, allowing observation of nutrient-dependent interactions with commensals. Experimental optimization, including media composition and preparation of seedlings and bacteria, is discussed. This system provides a framework that can be adapted to study plant-microbiota interactions in further nutritional contexts.

For complete details on the use and execution of this protocol, please refer to Harbort et al. (2020).

## Highlights

• A gnotobiotic system to reconstitute the plant microbiota and control iron availability
• Iron is present but unavailable due to basic pH, mimicking iron-limiting soils
• Identification of beneficial plant commensals that rescue iron-limiting plant growth
• System allows study of nutrition-dependent plant-microbiota interactions

## Graphical Abstract

**Figure:**
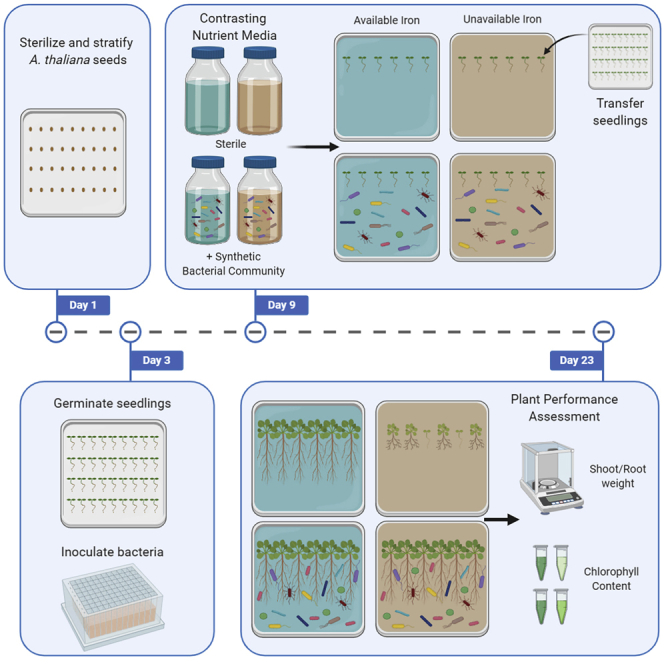

## Before you begin

In this protocol, we will assess *Arabidopsis thaliana* accession Col-0 performance when grown axenically or in the presence of a synthetic community (SynCom) of bacteria on two media with contrasting iron availability: a replete, “available iron” condition and an iron-limiting “unavailable iron” condition. In this system, the concentration of iron is identical in both media (100 μM); the availability is varied by providing forms of iron that differ in their solubility at high pH. By buffering the media strongly at pH 7.4 with 10 mM HEPES, we mimic iron-limiting calcareous soils, where iron is present but unavailable to plants (Kobayashi and Nishizawa, 2012). In the “unavailable iron” condition, iron(III) (supplied as FeCl_3_) is sparingly soluble and is unavailable to plants. This is critical for assessing nutritionally beneficial host-microbiota interactions, which are precluded in artificial systems where iron is removed altogether (Harbort et al., 2020). As a control “available iron” medium with the same iron content and pH, the media is supplied with the same concentration of a chelated form of iron (FeEDTA, Ferric sodium ethylenediaminetetraacetate), which has increased solubility even in these alkaline conditions. To assess the impact of bacterial commensals on host performance in these conditions, the media are inoculated with a SynCom of rhizobacterial strains originally isolated from *Arabidopsis* roots grown in soil (Bai et al., 2015). Rhizobacterial culture collections of different origins can also be employed and compared. The complexity and diversity of the SynCom can be adjusted to suit the experimental question, or reduced to single strains in mono-associations.

In this protocol, surface-sterilized seedlings are pre-germinated for 5–6 days on axenic growth medium containing vitamins and sucrose, and then transferred to experimental conditions with controlled iron availability and commensals. Pre-germination and seedling transfer improve seedling germination rate and synchrony, which can vary between seed batches and genotypes, reducing variation within each condition.

### Prepare stock solutions

**Timing: ∼2 h**

1. Prepare 1/2 Murashige and Skoog (MS) media stock solutions
  a. Prepare stock solutions for MS media preparation according to Table 1.

**Table 1:** Stock solutions and recipe for 1/2 MS media

Stock solutions	Component	Molecular weight	Stock concentaration (mM)	g/L stock preparation	Final concentration (μM)	Stock volume for 1 L 1/2 MS (mL)
1	MgSO_4_·7H_2_O	246.48	30	7.3944	750	25
2	KH_2_PO_4_	136.09	25	3.40225	625	25
3	NH_4_NO_3_	80.04	1,000	80.04	10,000	10
4	KNO_3_	101.1	940	95.034	9,400	10
5	CaCl_2_·2H_2_O	147.01	150	22.0515	1,500	10
6	CoCl_2_·6H_2_O	237.93	0.053	0.01261029	0.053	1
7	CuCl_2_·2H_2_O	170.48	0.05	0.008524	0.05	1
8	H_3_BO_3_	61.83	50	3.0915	50	1
9	KI	166	2.5	0.415	2.5	1
10	MnCl_2_·4H_2_O	197.91	50	9.8955	50	1
11	Na_2_MoO_4_·2H_2_O	241.95	0.52	0.125814	0.52	1
12	ZnCl_2_	136.3	15	2.0445	15	1
13	KCl	74.55	940	70.077	9,400	10
14a	FeEDTA	367.05	100	36.705	100	1
14b	FeCl_3_.6H_2_O	270.3	100	27.03	100	1
  b. Dissolve solutions 1–13 in distilled deionized water (ddH_2_O), autoclave, and store at 20°C–22°C.
  c. Dissolve solution 14a in ddH_2_O, and 14b in 10 mM HCl. Filter sterilize these solutions and store at 4°C protected from light.
2. Prepare buffer stock solution (250 mL, final concentration 2 M)
  a. Weigh out 119.1 g HEPES powder (4-(2-hydroxyethyl)piperazine-1-ethanesulfonic acid, N-(2-Hydroxyethyl)piperazine-N′-(2-ethanesulfonic acid)) and ∼18 g KOH tablets.
  b. Slowly add HEPES to 100 mL ddH_2_O, intermittently adding KOH tablets. Wait until the last addition is mostly dissolved before adding more to prevent clumping. The KOH tablets release heat during dissolution, which helps the HEPES dissolve while bringing the solution to a basic pH.
  c. When everything has dissolved, adjust the volume to near 250 mL with ddH_2_O and bring the pH to 8.1–8.2 using a well-calibrated pH electrode. Adjust pH as needed with high concentration HCl or KOH. It is important that the HEPES is completely dissolved before proceeding, otherwise the pH will continue to change as it dissolves.
  d. Adjust final volume to 250 mL, sterile filter, and store at 4°C protected from light.
3. Prepare 10 mM MgCl_2_ by dissolving 2 g of MgCl_2_·6H_2_O in 1 L of ddH_2_O and autoclaving.

### Verify HEPES buffer strength and the final pH of MS media

**Timing: ∼1 h**

4. Prepare liquid unavailable iron medium by combining stock solutions 1–13, and 14b in volumes indicated in Table 1. This medium does not need to be sterilized, as it will only be used for pH adjustment.
5. To test pH and buffer strength of HEPES stock solution, combine 50 mL of 1/2 MS media with FeCl3 with 250 μL of HEPES solution (10 mM final) and mix. Measure the pH of the solution. The final pH should be 7.2–7.4 for iron limitation.
6. If the final pH is too high or too low, adjust the pH of the HEPES stock solution with KOH or HCl to compensate. Repeat step 5 to test the final pH with the adjusted HEPES solution. Repeat until the proper pH is obtained. Troubleshooting 2

**CRITICAL:** The concentration and pH of the HEPES solution are critical to the success of this assay. Close attention must be paid to obtaining a proper pH in the final media used for the assay with each batch of HEPES. Measuring the pH of the stock solution only is insufficient as the measurement is inaccurate in such a strongly buffered solution.

### Prepare seedling germination plates

**Timing: ∼5 h**

7. Prepare agar media for seed germination (1/2 MS with vitamins, 0.5% sucrose, 1 g/L MES pH 5.7).
  a. Per 1 L of ddH_2_O, dissolve 2.2 g of powdered MS medium containing vitamins, 5 g of sucrose, and 1 g MES. Once dissolved, adjust pH to 5.7.
  b. Add 10 g of low-impurity bacteriological agar and autoclave media. Allow autoclaved media to cool to a temperature that can be handled without solidifying (∼45°C).
  c. In a laminar flow hood, pour media into 120 mm × 120 mm square petri dishes by measuring ∼45 mL per plate in a 50-mL conical tube, then pouring into plates. Allow plates to dry with lids open for ∼30 min.
  d. Plates can be prepared in bulk and stored at 4°C for months.

***Note****:* For seed germination plates, we use a traditional 1/2 MS medium from powder complete with vitamins and sucrose to increase the germination rate and obtain synchronously grown seedlings. Media for experimental conditions are prepared using individual stock solutions to control iron availability.

### Prepare axenic seedlings

**Timing: 7 days**

**Figure 1:**
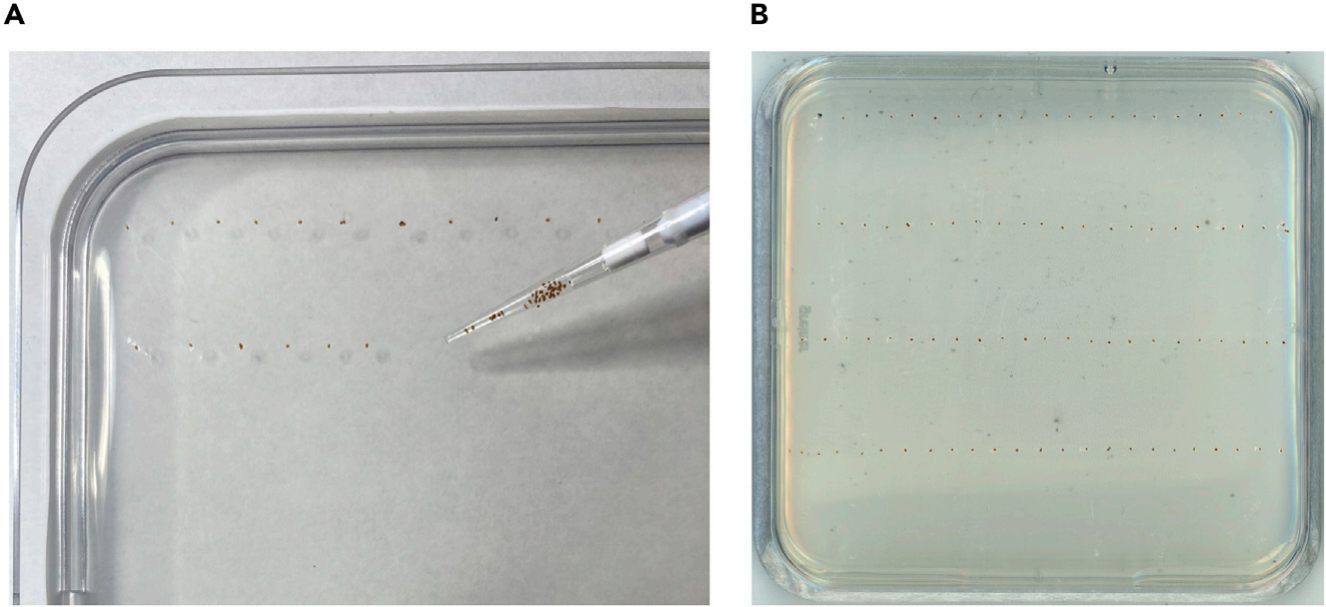
Placement of sterilized seeds onto germination media plates

8. Surface-sterilize *A. thaliana* seeds
  a. Measure out the desired number of seeds and transfer to a 2-mL microcentrifuge tube.
  b. Add 1.5 mL of 70% ethanol and incubate for 15 min with rotation or agitation.
  c. Working under sterile conditions, allow seeds to settle to the bottom of the tube and remove as much supernatant as possible.
  d. Wash seeds twice more with 70% ethanol, then once with 96% (v/v) ethanol.
  e. Remove as much ethanol as possible without aspirating seeds.
  f. Wash 5 times with sterile ddH_2_O to remove all traces of ethanol. Seeds can be pelleted by briefly centrifuging to facilitate water removal without removing seeds.
  g. Resuspend seeds in 1 mL ddH_2_O.
9. Transfer seeds onto germination plates (Figure 1).
  a. In a laminar flow hood, transfer seeds into rows on to plates containing germination agar media. Using a 20-μL pipette with a filter tip, aspirate seeds with some water, then deposit individual seeds in rows (∼25 seeds per row, 5–6 rows per plate). Having a small space between individuals makes transferring to experimental plates quicker and easier. Rows should be 2.5–3 cm apart to allow for root growth.
  b. Leave plates horizontal with lids open for a few minutes to allow residual water to dry or absorb into the agar matrix.
  c. Close plates, seal with micropore tape, and store for 48 h at 4°C in the dark to stratify seeds.
  d. Transfer plates to a light chamber standing vertically with the program: 10 h light, 60–70 PPFD (μmol m^−2^ s^−1^) at 21°C/14 h dark, 19°C. These plant growth conditions are used for the entire protocol.
  e. Allow seeds to germinate and grow for 5–6 days. Troubleshooting 1

### Grow bacterial cultures

**Timing: 1–7 days**

10. Inoculate bacterial cultures
  a. Timing for this step will depend on the bacteria to be used and the growth conditions. Different taxa of bacteria grow at different rates. It also makes a difference if bacteria are cultured in 96-well format deep-well plates (for large number of strains, but grow more slowly), or test tubes (for fewer strains, but allows for faster growth). Allow bacteria to grow to late exponential growth phase.
  b. For microbiota reconstitution with a taxonomically diverse synthetic community (SynCom), inoculate cultures in deep-well plates in appropriate nutrient medium (e.g., 1/2 TSB medium) and seal with micropore film to allow aeration. Culturing in 96-well format enables faster experiment setup when using many strains.
  c. Grow SynCom strains for 5 days at 25°C with shaking, until even slow-growing strains have significant growth.
  d. One day before experiment setup, resuspend strains by pipetting, dilute 1:100 into fresh medium, and grow 15–18 h at 25°C. Keep the original cultures growing as well. This ensures that both fast- and slowly growing strains are present in exponential growth phase.
  e. Alternatively, if growing fewer strains in test tubes, cultures can be subcultured 15–18 h, or for a few hours before experiment setup.

### Prepare experimental media

**Timing: ∼3 h**

11. Prepare 1/2 MS media for contrasting iron conditions. Media can be made the evening before experiment set up, stored at 4°C, and autoclaved first on the setup day.
  a. Calculate the amount of medium needed.
    i. We recommend at least three replica plates per condition. Calculate 50 mL of medium per plate.
  b. Prepare base MS medium (without iron or HEPES buffer) from stock solutions with 1% agar. At this point, both available iron (FeEDTA) and unavailable iron (FeCl_3_) media are identical and can be made in one batch.
    i. Refer to Table 1 for calculating the amount of each stock solution needed to make base media.
    ii. Combine appropriate amounts of solutions 1–13 and dilute with ddH_2_O to final volume needed.
    iii. Separate into glass bottles for available and unavailable iron media. Add 1% agar, autoclave, and keep warm (50°C) to prevent solidification until use.

**CRITICAL:** Add a magnetic stir bar to each bottle of base media before autoclaving to ensure proper mixing of added components during experimental setup.

## Key resources table

**Table:** 

REAGENT or RESOURCE	SOURCE	IDENTIFIER
**Bacterial and virus strains**		

AtSphere *Arabidopsis thaliana*-derived culture collection	Bai et al., 2015	http://www.at-sphere.com/

**Chemicals, peptides, and recombinant proteins**		

MgSO_4_·7H_2_O	Sigma-Aldrich	CAT#M2773
KH_2_PO_4_	Merck	CAT#104873
NH_4_NO_3_	Sigma-Aldrich	CAT#A9642
KNO_3_	Merck	CAT#1.05063
CaCl_2_·2H_2_O	Sigma-Aldrich	CAT#449709
CoCl_2_·6H_2_O	Sigma-Aldrich	CAT#255599
CuCl_2_·2H_2_O	Sigma-Aldrich	CAT#C3279
H_3_BO_3_	Sigma-Aldrich	CAT#B6768
KI	Sigma-Aldrich	CAT#221945
MnCl_2_·4H_2_O	Sigma-Aldrich	CAT#M5005
Na_2_MoO_4_·2H_2_O	Sigma-Aldrich	CAT#M1003
ZnCl_2_	Sigma-Aldrich	CAT#Z0152
KCl	Sigma-Aldrich	CAT#1.04936
FeEDTA	Sigma-Aldrich	CAT#E6760
FeCl_3_ hexahydrate	Merck	CAT#103943
Murashige and Skoog (MS) medium with vitamins	Duchefa Biochemie	CAT#M0222
Agar Bacteriological	Difco	CAT#214530
HEPES buffer	Roth	CAT#6763.1

**Deposited data**		

Example data and R scripts for analysis	Harbort et al., 2020	Mendeley Data https://doi.org/10.17632/tkdn6zbw7k.1

**Experimental models: organisms/strains**		

*A. thaliana*: Col-0 wild type	NASC	N60000

**Software and algorithms**		

BioRender (graphical abstract)	BioRender.com	RRID: SCR_018361
R statistical environment	https://www.r-project.org	V 4. 0. 1

## Materials and equipment

**CRITICAL:** Solution 14b (FeCl_3_) is prepared in 10 mM HCl to prevent precipitation; all other components are dissolved in ddH_2_O. Solutions 1–13 can be sterilized by autoclaving and stored at 20°C–22°C. Solutions 14a and 14b should be filter sterilized and stored at 4°C protected from light.

## Step-by-step method details

### Prepare SynCom for inoculation

**Timing: 1–2 h**

Wash bacteria to remove antibiotics and other metabolites potentially in the culture media, and then combine into a SynCom for inoculation into MS agar media.

1. Wash and resuspend bacterial cultures.
  a. Centrifuge bacteria at 4,000 × *g* for 15 min.
  b. Carefully remove supernatant without disturbing bacterial pellet using a pipette.
  c. Resuspend pellets in an equal volume of 10 mM MgCl_2_.
  d. For SynCom experiments: combine strains in a conical tube, centrifuge again at 4,000 × *g* and resuspend well in 10 mM MgCl_2_.
2. Resuspend bacterial cultures to desired OD_600_.
  a. Measure OD_600_ of bacterial suspension using a photometer. Be sure to measure a dilution of the suspension within the linear range of your photometer.
  b. Dilute SynCom mixture to OD_600_ = 0.1 in 10 mM MgCl_2_. This stock solution will be used to inoculate growth media. Troubleshooting 3
3. For a heat-killed negative control, incubate an aliquot of bacterial inoculant in a heat block at 99°C for 20 min.

**CRITICAL:** For complex SynComs with many strains growing in 96-well plates, both the original culture plate and the subcultured plate are combined to capture both slow and fast-growing strains in growth phase. Ensure that all bacteria are pelleted during the centrifugation step. Depending on the bacteria being used, you may need to adjust the centrifugation time. For complex SynComs it is generally not feasible to adjust the OD_600_ of each strain individually to control the ratios of strains in the input. When working with fewer strains, as in mono-associations, it may be desirable to adjust the OD_600_ of each strain before combining. Bacteria are resuspended at OD_600_=0.1, to be used as 1,000× dilution in media (Final OD_600_=0.0001, ∼10^4^–10^5^ CFU/mL). This may need to be adjusted for individual bacteria or experimental purposes.

### Pour experimental media plates

**Timing: 2–3 h**

Media are supplied with available or unavailable forms of iron, inoculated with bacteria, and poured into plates.

4. Add iron and pH buffer to base MS media
  a. Allow autoclaved base MS media (from step 11 above) to cool to ∼50°C.
  b. Place media bottle on a magnetic stir plate in a laminar flow hood. Stir at medium speed and add appropriate volume of FeEDTA or FeCl_3_ solution (stocks are 1,000×).
  c. Continuing to stir, add necessary volume of HEPES buffer (stock is 200×).
  d. Allow media to cool in water bath to 42°C–45°C. Troubleshooting 3
5. Pour experimental plates
  a. For each plate, 45 mL of media will be poured into a square 120 × 120 mm petri dish.
  b. Add 50 μL of bacterial solution, or heat-killed SynCom negative control, to the bottom of a 50-mL conical tube (for a 1:1,000 dilution). Pour 45 mL of prepared and cooled media into the tube gently to prevent bubbles. Close lid and invert several times to mix.
  c. Pour media into petri dish and allow to dry with lid open for 20 min.

***Note****:* Once HEPES buffer is added, media should be intermittently stirred on a magnetic stir plate to ensure equal distribution of iron without precipitation.

**Pause Point:** Experiment can be paused after drying plates for up to several hours before continuing to seedling transfer. After drying, cover plates with lids and leave until ready to continue.

### Transfer plant seedlings

**Timing: 1–3 h**

**Figure 2:**
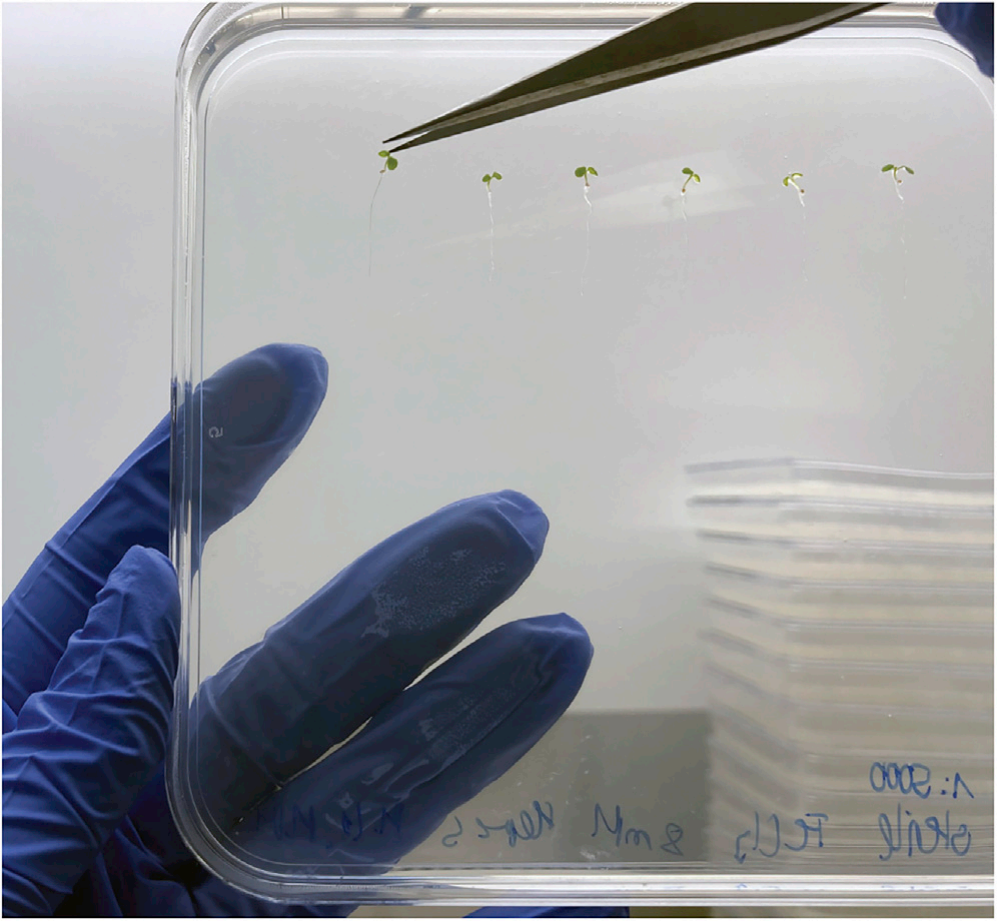
Transfer of seedlings onto experimental media plates

Axenic seedlings are transferred from germination plates to experimental media plates (Figure 2).

6. Visually check germination plates for signs of contamination, which should be apparent by day 5.
7. To transfer, gently remove seedlings from the plate by lifting under cotyledons with a sterile pipette tip or forceps. Avoid damaging the seedlings by overhandling or pinching.
8. Place seedlings (6–8 per plate) in a row near the top of the experimental plates, leaving enough room for shoot growth.
9. Seal plates with micropore tape and place vertically in a light chamber with the program: 10 h light, 60–70 PPFD (μmol m^−2^ s^−1^) at 21°C/14 h dark, 19°C.
10. Grow for 2 weeks, rotating the position of the plates within the growth chamber every 2–3 days to prevent location effects.

***Note****:* Discard tips used for seedling transfer frequently to avoid contaminations. Always use a new tip when transferring to sterile control plates. Alternatively, if using forceps, sterilize between plates with 70% ethanol.

### Evaluate plant performance

**Timing: 3–5 h**

After 2 weeks of growth, evaluate plant performance by shoot fresh weight and chlorophyll measurement.

11. Using a scalpel, cut through micropore tape seal and open plates.
12. To measure shoot fresh weight (SFW), cut seedlings at the shoot-root junction, being careful not to cut off any leaves. Remove plant shoot with forceps and blot on paper towels to remove from the leaves any water condensation. Record weight of individual plant shoots using a precision scale. Dead plants are recorded as 0.
13. Measure chlorophyll content of a pooled leaf sample from each plate.
  a. After measuring the fresh weight of all plants from a single plate, collect a single leaf from each plant into a 2-mL microcentrifuge tube. Measure tube weight before and after adding sample to record input weight. This should be 20–30 mg of tissue.
  b. Freeze chlorophyll samples in liquid nitrogen and store at −80°C until further analysis.
  c. Extract chlorophyll by adding 1 mL DMSO to sample tubes and incubating with shaking at 65°C for 45–60 min, until leaf samples are clear and extracted. If the input sample weight was ≥30 mg, increase the amount of DMSO added to compensate.
  d. Measure the absorbance at 652 nm of the DMSO extract using a spectrophotometer.
  e. Calculate the chlorophyll concentration in the extract with the formula (Hiscox and Israelstam, 1979):
    ▪ Chlorophyll mg/L = Abs_652_ ∗ (1000/34.5)
  f. Multiply the chlorophyll concentration in the extract by the volume of DMSO added (in mL), and divide by the input sample weight (in g) to obtain the plant chlorophyll content (mg/g of fresh weight).

## Expected outcomes

**Figure 3:**
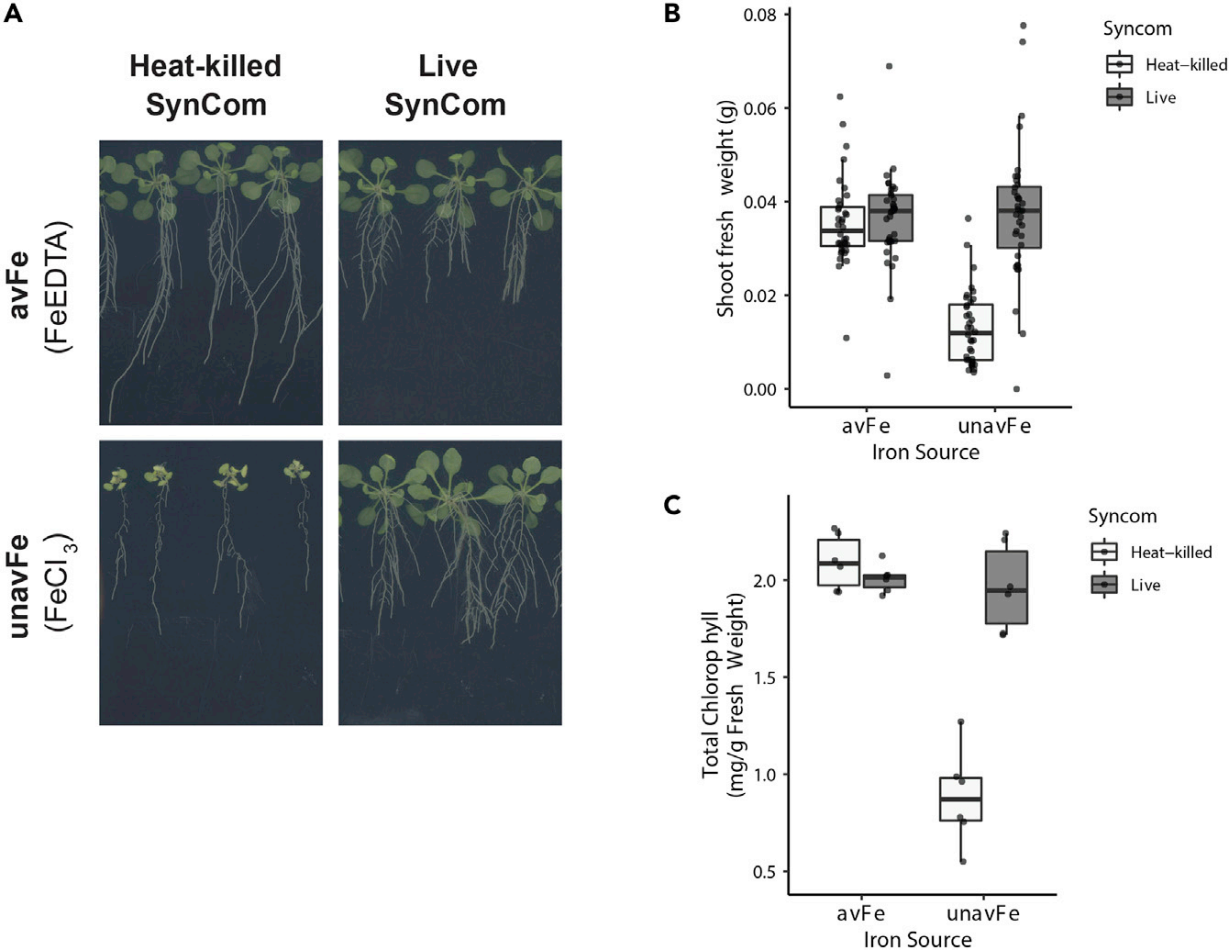
Representative protocol results with Col-0 wild-type plants and diverse SynCom (A) Representative images of plants after 2 weeks of growth on experimental conditions. (B and C) (B) SFW and (C) chlorophyll quantification (n = 36 plants, 6 pooled leaf chlorophyll samples per condition).

After 2 weeks on experimental plates, there should be clear differences in axenic plant growth between available and unavailable iron conditions (Figure 3). Plants grown on unavailable iron conditions should be smaller, with shorter roots, and noticeably chlorotic (yellow) leaves compared to plants on available iron. Bacteria-inoculated plants will have variable phenotypes, depending on the bacteria and plant genotype. Often a small proportion of plants will not survive the experiment, especially in iron-limiting media. These plants should be included in the results and recorded as zero values.

## Quantification and statistical analysis

For statistical analysis, we recommend performing experiments with three replica plates per condition, each containing 5–6 individual plants (n = 15–18 plants per experiment). Shoot fresh weight should be plotted with boxplots or other appropriate methods to visualize individual data points (single plants) and visualize data dispersion. Chlorophyll content should be normalized to sample input weight and plotted as fresh weight showing individual data points (pooled leaf samples) and data dispersion. For statistical analysis of plant weight data, nonparametric tests – such as a Kruskal-Wallis, Wilcoxon signed-rank test, and/or Dunn’s pairwise comparison test – are preferred as data are usually not normally distributed, especially when zero values are present. Leaf chlorophyll content usually shows approximately normal distribution and should be analyzed with ANOVA and Tukey’s HSD.

## Limitations

This assay provides a model for studying the impact of bacterial commensals and iron deficiency on plant performance. Especially relevant is the mechanism of iron limitation, which mimics conditions in iron-limiting alkaline soils where iron is present but unavailable to plants. However, as with all experimental models, some limitations should be considered during the interpretation of results.

In this protocol, plants are grown for 5–6 days axenically before being transferred to media containing microbes. This does not reflect conditions in nature, where colonization of commensals would begin immediately upon germination. The importance of interactions with commensals during this early phase of root microbiota establishment may be significant. Similarly, plants are exposed to iron-limiting growth media after pre-germination on media in which iron is readily available instead of immediately upon germination. Iron-limiting conditions may also impact the process of seed germination significantly, which is overlooked in this assay. Furthermore, as plants are grown in petri dishes on an agar matrix, the full life cycle and reproductive success of the plant cannot be assessed due to space limitations and high humidity. Results in this reductionist system should be cautiously extrapolated to conditions in soils, as it does not reproduce the full chemical and biological diversity of soil or soil physical parameters. When coupled with experiments in nutritionally characterized soils for phenotype confirmation, this assay provides a robust and amenable method to studying plant-microbiota interactions in the context of iron deficiency.

## Troubleshooting

### Problem 1: contamination

Visible bacterial or fungal contamination develops on seedlings during germination

### Potential solution

Surface sterilization of seeds prior to germination is unsuccessful. Some seed batches may be contaminated with microbes or spores that are more difficult to eliminate. Several adjustments can be made to improve sterilization efficiency (Lindsey et al., 2017):

• Longer incubation times in 70% ethanol
• Sterilization with a solution containing bleach and Tween 20
• Sterilization with chlorine gas

### Problem 2: plants do not survive or grow on iron-limiting media

Many, or all, plants grown on iron-limiting media (FeCl_3_) fail to grow, or are dead by the end of the experiment

### Potential solution

The pH-induced iron limitation may be too severe. The buffer concentration or final pH of the media may be too high for plants to overcome. Measure the final pH of the agar media by cutting out a section of agar, shaking in twice the volume of ddH_2_O for 30 min, and measuring the pH. Ensure that the pH is not above 7.4. Adjust the pH of the HEPES stock solution if necessary, and again check the pH of final media. Alternatively, slightly reduce the amount of HEPES stock added if your stock is too concentrated or alkaline.

### Problem 3: large variation in plant growth

Large variation in plant growth within plates or among experimental groups

### Potential solution

This could arise from multiple potential sources.

• Seedling transfer
  ○ Be sure to transfer only germinated and growing seedlings to experimental plates. It is normal that a small portion of seeds do not germinate or seedling growth ceases after germination. These should be discarded and not transferred to experimental plates.
  ○ Be careful not to injure seedlings during transfer, as this will impact plant growth. If injury of a seedling is suspected during transfer, discard it.
• Insufficient bacterial inoculation
  ○ Ensure that the media is cooled to below 45°C before adding bacteria. If not properly cooled, the heat may kill the bacteria.
  ○ Check the final OD_600_ of your bacterial inoculant is 0.1. If this problem persists, the dose of inoculum can be increased.

## Resource availability

### Lead contact

Further information and requests for resources and reagents should be directed to and will be fulfilled by the Lead Contact, Paul Schulze-Lefert (schlef@mpipz.mpg.de).

### Materials availability

This study did not generate new unique reagents. All bacterial strains and *A. thaliana* lines used in this study were previously described and are publicly available.

### Data and code availability

Example datasets and scripts for analysis from (Harbort et al., 2020) have been deposited to Mendeley Data with the https://doi.org/10.17632/tkdn6zbw7k.1.
